# Iterative Positioning Algorithm for Indoor Node Based on Distance Correction in WSNs

**DOI:** 10.3390/s19224871

**Published:** 2019-11-08

**Authors:** Jing Chen, Shixin Wang, Mingsan Ouyang, Yuting Xuan, Kuan-Ching Li

**Affiliations:** 1 School of Electrical and Information Engineering, Anhui University of Science and Technology, No.168, Taifeng Road, Huainan 232001, China; jchen@aust.edu.cn (J.C.); 2018200560@aust.edu.cn (S.W.); msoy@aust.edu.cn (M.O.); 2018200572@aust.edu.cn (Y.X.); 2 Department of Electrical Engineering and Electronics, University of Liverpool, Liverpool L69 3BX, UK; 3 Department of Computer Science and Information Engineering (CSIE), Providence University, Taichung 43301, Taiwan; 4 Hubei Education Cloud Service Engineering Technology Research Center, Hubei University of Education, Wuhan 430205, China

**Keywords:** iterative positioning algorithm, distance correction, RSSI, noise impact factor, distance deviation coefficient

## Abstract

Node position information is critical in wireless sensor networks (WSN). However, existing positioning algorithms commonly have the issue of low positioning accuracy due to noise interferences in communication. Hence, proposed in this paper is an iterative positioning algorithm based on distance correction to improve the positioning accuracy of target nodes in WSNs, with contributions including (1) a log-distance distribution model of received signal strength indication (RSSI) ranging which is built and from which is derived a noise impact factor based on the model, (2) the initial position coordinates of the target node obtained using a triangle centroid localization algorithm, via which the distance deviation coefficient under the influence of noise is calculated, and (3) the ratio of the distance measured by the log-distance distribution model to the median distance deviation coefficient which is taken as the new distance between the target node and the anchor node. Based on the new distance, the triangular centroid positioning algorithm is applied to calculate the coordinates of the target node, after which the iterative positioning model is constructed and the distance deviation coefficient updated repeatedly to update the positioning result until the criteria of iterations are reached. Experiment results show that the proposed iterative positioning algorithm is promising and effectively improves positioning accuracy.

## 1. Introduction

Wireless sensor networks have been widely applied in indoor scenarios where satellite or cellular signals do not work properly, primarily in fields of defense, industry, and social life due to the networks’ advantages of low power consumption, low cost, and self-organization [1]. In order to provide adequate monitoring services in engineering applications, node position information must be provided [2,3]. Node position information is the key to whether the information obtained is valuable or not in the WSN-based Zigbee protocol, especially for target surveillance and tracking in the fields of military work and anti-terrorism [4,5,6,7]. That is, it can be said that perceived data is meaningless if no node position information is provided. However, wireless signals are inevitably interfered with by noises such as multi-path fading [8,9], diffraction [10], antenna gain [11], and non-line of sight [12] when propagating in actual physical environments, and uncertain propagation loss is produced, resulting in inaccuracies in ranging. It is known that the maximum ranging error rate is up to ±50% [13].

To solve this problem, issues and methods on node positioning in WSN should be widely explored. An effective node positioning method must consider the following points. (1) How should a mathematical model be constructed to fit the nonlinear relationship between the received signal strength indication (RSSI) and the distance? (2) What positioning algorithm should be used to obtain a higher positioning accuracy? (3) What are the basic requirements to consider in terms of hardware resources and computational complexity when building a positioning algorithm?

**Figure 1:**
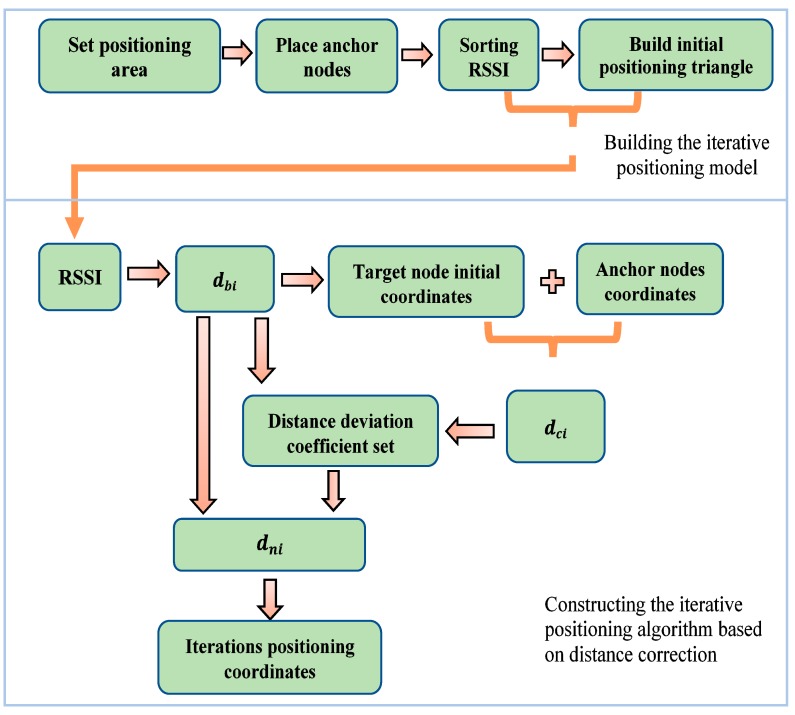
The process of our target node iterative positioning model based on distance correction. The *d_bi_* is the distance between the given anchor node and unknown target node, which is calculated by the log-distance distribution model in Figure 1. The *d_ci_* is the distance between the coordinates of the anchor node and the coordinates calculated by the triangle centroid positioning algorithm. The *d_ni_* is the distance between the anchor node and the target node, which is recalculated based on the median. Legend: RSSI, received signal strength indication.

Based on the three issues mentioned above, we propose a target node iterative positioning algorithm based on distance correction in this paper. The motivation of this paper is to reduce the positioning error of the target node to support users to retrieve accurate position information. The process of our target node iterative positioning model based on distance correction is shown in Figure 1.

In the proposed algorithm, the median of the distance deviation coefficients is used to modify the measured distance during each iteration. The median of the distance deviation coefficients can more closely express the overall distance deviation characteristic. The log-distance distribution model is applied to calculate the distances among unknown target nodes and connected anchor nodes to fit more accurately the nonlinear relationship between distance and RSSI, reducing computational complexity as well. The iterative positioning model is constructed to ensure that the target node is in the region surrounded by its connected anchor nodes, which is also of great help to improving the positioning accuracy. Additionally, the node iterative positioning algorithm based on distance correction can provide theoretical support for future research. Contributions are included in the following aspects.

(1) Derivation of noise impact factor based on a log-distance distribution model
  The expression of the noise impact factor *F_N_* is derived by reconstructing the mathematical model, which is the corresponding numerical relationship between the noise impact factor *F_N_* and the measured distance. The noise impact factor derived in this paper is used to describe the influence degree of noise on the measured values of RSSI.
(2) The selection of distance deviation coefficient on node measured distance
  The distance deviation coefficient is used to evaluate the deviation degree of the distances calculated by the log-distance distribution model and the triangle centroid algorithm, respectively, and a distance deviation coefficients set is established. The median of the distance deviation coefficient set is selected to characterize the measured distance deviation of all nodes, which can better reflect the overall distance deviation characteristic.
(3) Construction of the iterative positioning algorithm based on distance correction
  The distance deviation coefficient median is used as an iteration factor for the iterative positioning algorithm. In the process of each iteration, the median of the distance deviation coefficients is used to correct the distance from the last positioning, obtaining a distance value closer to the real value. The triangle centroid localization algorithm is iteratively applied to reduce the positioning fluctuation error and improve positioning accuracy.

The rest of this paper is organized as follows. A brief review of related works is presented in Section 2, while the proposed iterative positioning algorithm is introduced in Section 3. The iterative positioning model is constructed in Section 4, the experimental results and corresponding analyses are given in Section 5, and, finally, conclusions and general discussions are summarized in Section 6.

## 2. Related Work

This section reviews two existing works that are the reference foundation theories for the positioning of the target node in WSN. The former is the measurement of the distance between the target node and the anchor node while the latter is to determine the position coordinates of the unknown target node.

### 2.1. The Measurement of the Distance between the Target Node and the Anchor Node

The measurement of the distance between the target node and the anchor node is an important research topic of the node positioning in WSN. Nowadays, most of the existing node positioning algorithms can be divided into two categories according to whether distance measurements are required or not. One is the range-based measurement positioning algorithm and the other is a range-free measurement positioning algorithm [14]. The distance measurement refers to calculating the distance between the unknown target node and the given anchor node connected to it through communication between them [15,16]. The classic distance measurement algorithms include the algorithm based-TOA (time of arrival), the algorithm based-TDOA (time difference of arrival) [17], the algorithm based-AOA (angle of arrival) [18] and the algorithm based-RSSI [19,20].

In the above distance measurement algorithms, the first three algorithms (TOA, TDOA, and AOA) need to calculate the distance between the unknown target node and given anchor node accurately by using an algorithm with high complexity that requires not only additional hardware equipment but also excellent node energy consumption. All these significantly increase the communication cost and energy consumption of the positioning process. Hence, the algorithm based-RSSI is adopted to measure distance in the paper for fitting the principle of low power consumption and low cost.

### 2.2. The Target Node Positioning Algorithm

The target node positioning algorithm can be built in several ways. A triangle centroid positioning (TCP) algorithm based on the distance or relative angle information between the target node and the anchor node has been proposed in the existing documents [21,22,23]. Since the node distribution characteristics are not fully considered, the problems in the TCP algorithm are twofold: the target node deviating active locating area and the significant positioning error. The weighted centroid positioning (WCP) algorithm has been presented in the existing literature [24,25,26,27] by introducing a weight factor, which is related to the distance estimation. Nevertheless, the positioning error will be significantly enlarged if there is a significant deviation in the distance estimating process of the WCP algorithm. A fingerprint database positioning algorithm has been constructed to improve the positioning accuracy in the existing literature [28,29,30] by collecting the positioning samples in advance. The positioning algorithm highly relies on fingerprint database data and the positioning accuracy will become weak if the environment changes. A positioning algorithm based on the neural network has been put forward in the literature where [31,32] the input of the algorithm is the value of RSSI and the output is the distance between nodes. Due to simple learning rules, the output of the neural network cannot be correct when the data is not sufficient. A positioning algorithm based on Bounding-Box has been proposed in the existing literature [33,34]. The number of given anchor nodes determines the positioning accuracy of the algorithm and the positioning accuracy is not high if the number of anchor nodes is not enough.

Aiming to address the above problems of low positioning accuracy, several experts have presented the solution of cooperative positioning. A cooperative positioning method fusing inertial-measurement unit and UWB ranging measurement is presented in [35] and a cooperative positioning method combining the inertial-measurement unit with long-range WiFi RSS and short-range UWB ranging measurements is proposed in [36] which significantly improves positioning accuracy. However, cooperative positioning methods need additional equipment. For positioning accuracy and low cost, an iterative positioning algorithm based on distance correction is proposed through correcting the estimated distance between the given anchor node and the target node and constraining the target node in the sub-triangular positioning area of the iterative positioning model in this paper. The algorithm calculates the target node coordinates iteratively and improves positioning accuracy effectively.

## 3. Iterative Positioning Algorithm for the Target Node Based on Distance Correction

An iterative positioning algorithm for the target node based on distance correction is proposed to help users overcome the influence of noise on positioning accuracy. The main issues presented in this paper for building an iterative positioning algorithm based on distance correction are threefold. First, a log-normal distribution mathematical model should be constructed to measure RSSI, based upon which the impact factor of noise on distance detecting can be derived. Second, a triangle centroid positioning algorithm should be built to determine the initial positioning coordinates of the unknown target node, and lastly, the distance deviation coefficient and its median can be determined. Therefore, the iterative positioning algorithm for the target node based on distance correction is constructed according to the median of the distance deviation coefficient.

### 3.1. Basic Concept

**Definition** **1.** *WSN node positioning. WSN node positioning refers to when the position of the unknown target node is calculated based on the communication between the anchor nodes whose position information is known in the network by specific techniques, algorithms, and schemes [37].*

**Definition** **2.** *Anchor node. The anchor node is the node whose coordinates or position information is known in WSN [38].*

**Definition** **3.** *Target node. The target node refers to the node whose coordinates or positioning information is unknown in the WSN [39].*

### 3.2. The Preprocessing of the Iterative Positioning Algorithm

Before the design of the iterative positioning algorithm, some pre-processing tasks must be performed. First of all, the mathematical model of the RSSI is constructed and used to calculate the initially measured distance. Then, the influence of noise on the measured distance is analyzed and thereby the noise impact factor is derived. Finally, the triangle centroid positioning algorithm is used to obtain the initial positioning coordinates of the target node.

#### 3.2.1. RSSI Ranging Algorithm

The concept of the RSSI ranging algorithm is to calculate the distance between the transmitting signal node and the receiving signal node by measuring the received signal strength, since there is a varying degree of losses in the propagating process of wireless signals. Therefore, it is imperative to build an appropriate RSSI ranging model. At present, the model used commonly is the log-distance distribution model [40,41,42,43].

*Pd* is used to indicate the power measurement corresponding to the distance between two nodes, denoted *d*. *Pd*_0_ is used to indicate the power measurement corresponding to the distance between two nodes, denoted *d*_0_. The relationship between *Pd* and *d* can be expressed as

(1) $$Pd=\frac{Pd_{0}}{{\left(d_{0}/d_{0}\right)}^{n}},$$

where *n* is a signal propagation factor, which is usually obtained by empirical value or actual calibration.

The logarithmic processing is performed on both sides of Equation (1), and after arranging, Equation (2) can be obtained as

(2) $$\mathrm{lg}Pd=\mathrm{lg}Pd_{0}-n\mathrm{lg}\left(\frac{d}{d_{0}}\right),$$

The relationship between RSSI value and power can be expressed as

(3) RSSI=10lgp,

Thus, a mathematical model of RSSI ranging can be obtained as

(4) $$P(d)=P\left(d_{0}\right)-10n\mathrm{lg}\left(\frac{d}{d_{0}}\right),$$

where *P*(*d*) is the RSSI value when the distance between two nodes is *d* and *P*(*d*_0_) is the RSSI value when the distance between two nodes is *d*_0_.

#### 3.2.2. The Effect of Noise on RSSI Ranging

The loss of wireless signals during propagation has a significant influence on the accuracy of the RSSI ranging algorithm and must be considered in practical applications. Next we will analyze the effect of signal propagation loss on RSSI ranging. In Equation (4), the measurement value *P*(*d*) is composed of the true signal strength value and the noise signal strength value, which are denoted *P_T_*(*d*) and *P_N_*(*d*), respectively. *P*(*d*_0_) is the RSSI value when the distance between the two nodes is *d*_0_. To simplify the calculation, *d*_0_ is usually taken as 1 and *P*(*d*_0_) is denoted as *A*.

Thus, the actual mathematical model of the log-distance distribution model is

(5) $$P_{T}\left(d\right)-P_{T}\left(d\right)=A-10n\mathrm{lg}\left(d\right),$$

From Equation (5), the distance between two nodes can be calculated as

(6) $$d=10^{\frac{A-[P_{T}\left(d\right)-P_{N}\left(d\right)]}{10n}},$$

Equation (6) can be calculated as follows by further mathematical transformation, i.e.,

(7) $$d=10^{\frac{A-P_{T}\left(d\right)}{10n}}10^{\frac{P_{N}\left(d\right)}{10n}},$$

Assume that $K_{1}=\frac{A-P_{T}\left(d\right)}{10n}$, $K_{2}=\frac{P_{N}\left(d\right)}{10n}$.

Equation (7) can be expressed as

(8) $$d=10^{K_{1}}\left(1+10^{K_{2}}-1\right),$$

Equation (8) can be expanded further as

(9) $$d=10^{K_{1}}+10^{K_{1}}(10^{K_{2}}-1),$$

Assuming that $d_{T}{=10}^{K_{1}}$, $F_{N}{=10}^{K_{2}}-1$, the calculation of distance *d* can be simplified to

(10) $$d=d_{T}+d_{T}F_{N},$$

where $d_{T}{=10}^{K_{1}}$ is the real distance between the given anchor node and unknown target node and $F_{N}{=10}^{K_{2}}-1$ is the impact factor of noise on distance measurement. *F_N_* is designated the noise impact factor.

#### 3.2.3. The Triangular Centroid Positioning Algorithm

**Figure 2:**
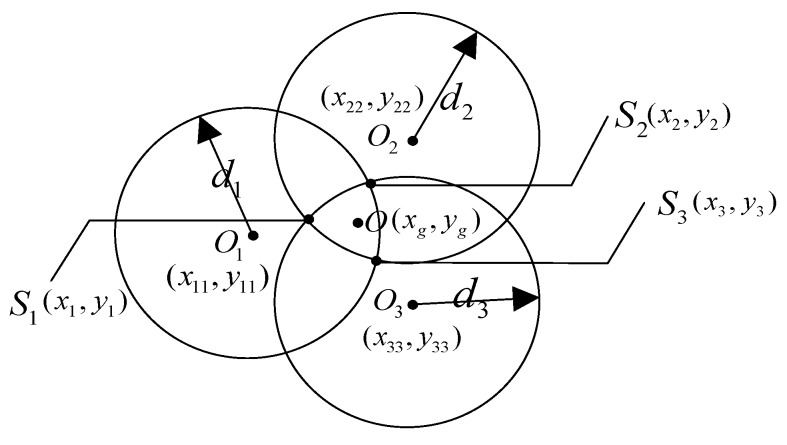
Schematic diagram of triangular centroid positioning.

The basic principle of the triangle centroid positioning algorithm is as follows: the three circles are determined by treating the three anchor nodes as their respective circle centers by addressing the distances between the given anchor nodes and unknown target node as their respective radiuses. The intersection of the three circles can obtain six intersection points and a triangle is constructed by treating the three closer intersection points as vertexes, with the centroid of a triangle taken as the coordinates of the node to be positioned. The schematic diagram of triangular centroid positioning is shown in Figure 2.

In Figure 2, *O*_1_, *O*_2_ and *O*_3_ are defined as the positions of three anchor nodes with coordinates of *O*_1_(*x*_11_, *y*_11_), *O*_2_(*x*_22_, *y*_22_), and *O*_3_(*x*_33_, *y*_33_) and whose radiuses are *d*_1_, *d*_2_, and *d*_3_, respectively. Points *S*_1_(*x*_1_, *y*_1_), *S*_2_(*x*_2_, *y*_2_), and *S*_3_(*x*_3_, *y*_3_) are the three closer intersections points, that is, the three vertices of the triangle centroid positioning algorithm.

The intersection point coordinates of the circles *O*_1_ and *O*_2_ can be obtained by Equation (11).

(11) $$\{\begin{array}{l} {\left(x-x_{11}\right)}^{2}+{\left(y-y_{11}\right)}^{2}=d_{1}^{2}\\ {\left(x-x_{22}\right)}^{2}+{\left(y-y_{22}\right)}^{2}=d_{2}^{2} \end{array},$$

In two sets of coordinates solved by Equation (11), the intersection S_3_ (*x*_3_, *y*_3_) is closer to the center of the positioning triangle. The solution of the remaining points *S*_1_(*x*_1_, *y*_1_) and *S*_2_(*x*_2_, *y*_2_) is similar to that of point *S*_3_(*x*_3_, *y*_3_).

Thus, the initial coordinates *O*(*x*_g_, *y*_g_) of the target node can be calculated by Equation (12).

(12) $$\{\begin{array}{l} x_{g}=\frac{1}{m}\sum_{i=1}^{m}x_{i}\\ y_{g}=\frac{1}{m}\sum_{i=1}^{m}y_{i} \end{array}\;(m=3),$$

where *m* = 3 and *i* = 1, 2, 3.

### 3.3. The Iterative Positioning Algorithm

In the actual physical environment, signals are easily disturbed by noise in the transmission process. Therefore, there is a large deviation between the distance obtained by the log-distance distribution model and the real distance value. To further reduce the positioning error, a node iterative positioning algorithm based on distance correction is introduced.

The basic principle of the iterative positioning algorithm based on distance correction is as follows: the distance deviation coefficient is introduced to evaluate the degree of deviation of the distance measured by the log-distance distribution model and the triangle centroid positioning algorithm, respectively. The distance between the given anchor node and the unknown target node is recalculated based on the distance deviation coefficient. By constantly updating the distance between the given anchor node and the unknown target node, the target node coordinates are iteratively calculated.

#### 3.3.1. The Calculation of the Distance Deviation Coefficient

The distance between the given anchor node and unknown target node, which is calculated by the log-distance distribution model, is denoted *d_bi_*. The distance between the coordinates of the anchor node and the coordinates calculated by the triangle centroid positioning algorithm is denoted *d_ci_*. To indicate the deviation of the two distances, the distance deviation coefficient *C_dev_* is defined by Equation (13).

(13) $$c_{dev}=\frac{d_{bi}}{d_{ci}}(i=1,2,3),$$

As distance deviation coefficients can be solved by Equation (13), it is essential to determine a characteristic quantity to represent the degree of deviation of the overall node measurement distance. The two statistical parameters, the average value and the median of distance deviation coefficients, can be closely used to express the overall distance deviation characteristics.

The average value of the distance deviation coefficients can express the average level of the overall measurement distance deviation. However, its fatal disadvantage is that if the extremum at both ends is too low or too high, the final calculation result will significantly deviate from the real situation.

Therefore, the median distance deviation coefficients are usually used to express the overall distance deviation characteristics. The median is not affected by the extreme values of both ends and can better reflect the overall distance deviation characteristics, making the final calculation result closer to the real situation.

The distance deviation coefficients are calculated by Equation (13). Then, the values of *C_dev_* are sorted to obtain the median named *C_m-dev_*.

The distance between the given anchor node and unknown target node can be recalculated based on the median *C_m-dev_*, as shown in Equation (14).

(14) $$d_{ni}=\frac{d_{bi}}{C_{m-dev}}(i=1,2,3),$$

The new distance *d_ni_* is obtained by Equation (14) and the triangular centroid positioning algorithm is iteratively conducted to obtain the positioning result (*x*_G_, *y*_G_).

#### 3.3.2. The Iteration Termination Criteria for the Algorithm

If the termination condition of the iterative positioning algorithm is set correctly, the higher positioning accuracy can be obtained within a small number of iterations and the algorithm is prevented from falling into an infinite loop. That is, the iteration termination condition is defined as

(15) $$P_{{\hat{O}}_{n}}-P_{{\hat{O}}_{n-1}}<\varepsilon ,$$

where $P_{{\hat{O}}_{n}}$ is the RSSI value between the centroid of the *n*th iteration and the unknown target node, and *ε* is the set threshold.

Under different environmental conditions, as Equation (15) is used as the iterative termination condition, its computational complexity is very high, even higher than the complexity of the iterative positioning algorithm itself. In this way, the hardware complexity of the system increases significantly and the algorithm becomes almost infeasible.

**Figure 3:**
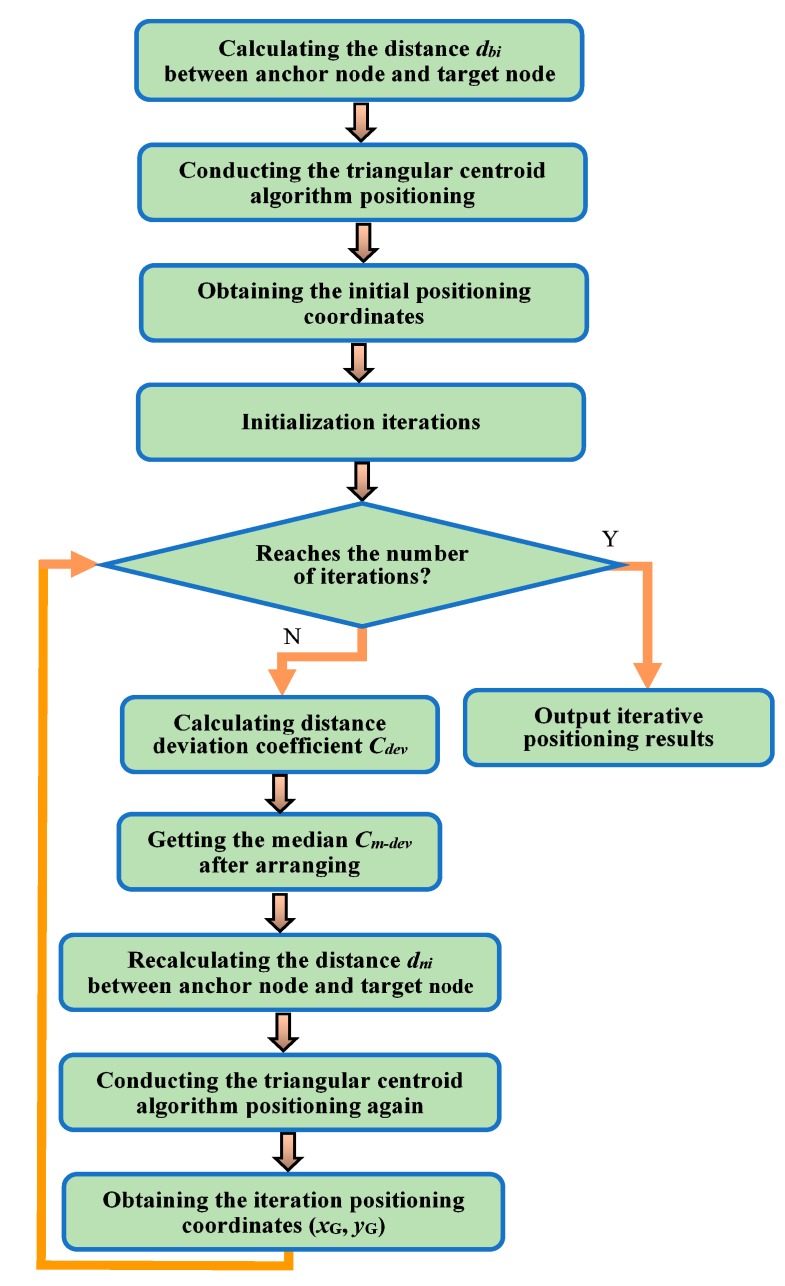
A flow chart of the iterative positioning algorithm.The *C_dev_* is the distance deviation coefficient in Figure 1. The *C_m-dev_* is the median of the distance deviation coefficients *C_dev_*.

The purpose of iterative positioning is to make the positioning error converge to the expected value, as the convergence property and convergence velocity are essential issues of the iteration. In the process of iteration, with the increased number of iterations, the convergence processes in which the error converges to the expected value usually have the following cases: fast convergence, slow convergence, and periodic oscillation. In order to further study the convergence property of the above three cases, a series of simulations are conducted to analyze the relationship between the positioning error and the number of iterations in the paper. Through simulation analysis, it can be noted that the iterative error varies with the above different regular patterns when the number of iterations is between 0–10 since the iteration error no longer varies when the number of iterations is between 10–20. A flow chart of the iterative positioning algorithm is shown in Figure 3.

## 4. The Construction of the Iterative Positioning Model

**Figure 4:**
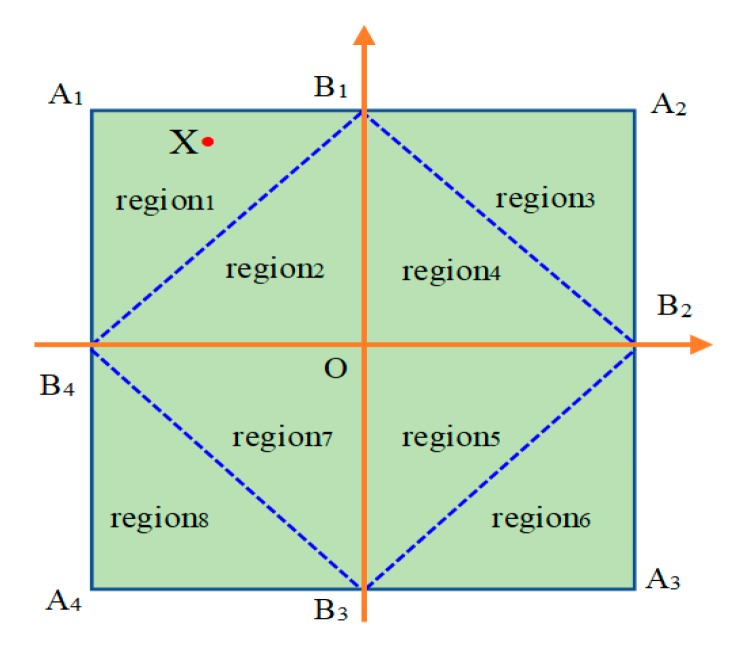
A schematic of the positioning model.

In the procedure of iterative positioning, the unknown target node in the positioning triangle area has a significant influence on its positioning accuracy. The positioning error of the target node located in the positioning triangle area is much smaller than that of the target node outside the positioning triangle area. To improve the positioning accuracy, an iterative positioning model is established in the paper, as shown in Figure 4.

In Figure 4, the quadrilateral *A*_1_*A*_2_*A*_3_*A*_4_ is a square, the point *O* is its center, and the points *B*_1_, *B*_2_, *B*_3_, and *B*_4_ are the midpoints of the respective sides. According to the connection shown in Figure 4, the square *A*_1_*A*_2_*A*_3_*A*_4_ is subdivided into eight triangular regions: region 1 to region 8. The anchor nodes (9 in total) are placed at points *A*_1_, *A*_2_, *A*_3_, *A*_4_, *B*_1_, *B*_2_, *B*_3_, *B*_4_, and *O*.

There is a target node *X* in the quadrilateral *A*_1_*B*_1_*OB*_4_ in Figure 4 and the points closest to the point *X* are points *A*_1_, *B*_1_, *O*, and *B*_4_ in turn. The node X is included in both Δ*A*_1_*B*_1_*B*_4_ and Δ*A*_1_*B*_1_*O*. In Δ*A*_1_*B*_1_*B*_4_, the coordinates (*x*_G1_, *y*_G1_) are calculated by the iterative positioning algorithm. In order to reduce effectively the positioning error caused by noise, the other coordinate (*x*_G2_, *y*_G2_) is calculated by the iterative positioning algorithm in Δ*A*_1_*B*_1_*O*. The weighted average of these two coordinates can be taken as the final coordinates (*x*_G3_, *y*_G3_). The closer the distance between the given anchor node and the unknown target node is, the more reliable the calculation result is. Hence, the weight of the former coordinate should be higher than the weight of the latter coordinate.

Simulation experiments compare the various proportion of weight values and the results show that when the weight of the former coordinate is 0.75 and the weight of the latter coordinate is 0.25, the positioning result is better than others.

The final positioning results can be expressed as follows in Equation (16).

(16) $$\{\begin{array}{l} x_{G3}=0.75x_{G1}+0.25x_{G2}\\ y_{G3}=0.75y_{G1}+0.25y_{G2} \end{array},$$

In actual positioning progress, the RSSI values of the nine anchor nodes are recorded and arranged in descending order. The first four larger *RSSI* values are denoted *RSSI*_1_, *RSSI*_2_, *RSSI*_3_, and *RSSI*_4_. The primary positioning coordinate (*x*_G1_,*y*_G1_) is calculated by *RSSI*_1_, *RSSI*_2_, *RSSI*_3_ and their corresponding coordinates. The second positioning coordinate (*x*_G2_,*y*_G2_) is calculated by *RSSI*_1_, *RSSI*_2_*, RSSI*_4_, and their corresponding coordinates. The weighted average of two coordinates according to the weights mentioned above is the final positioning result (*x*_G3_,*y*_G3_).

**Figure 5:**
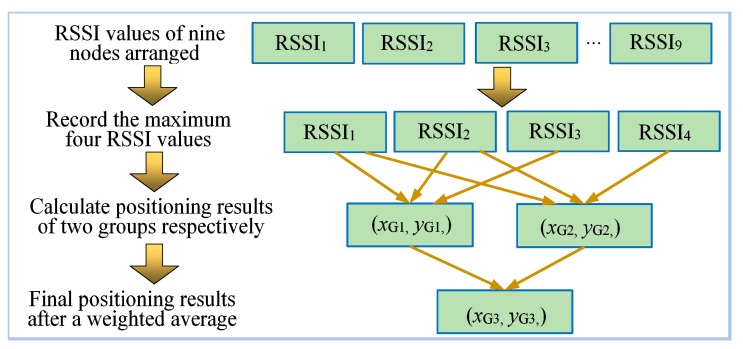
The specific implementation process of iterative positioning.

The positioning process of the iterative positioning algorithm is shown in Figure 5.

## 5. Experimental Results

### 5.1. The Method

To accurately and quantitatively verify the performance of the iterative positioning algorithm proposed, the experimental positioning area is set as a square of 40 m × 40 m with nine anchor nodes located at the vertices, center, and in the middle of the edges of the square. Now, 50 target nodes are generated in the square area by random and their positioning results calculated in turn.

In this experiment, for different noise impact factors *F_N_*, positioning error is discussed by three methods, namely, the centroid positioning algorithm, the weighted centroid positioning algorithm, and the iterative positioning algorithm based on distance correction. The value of *F_N_* is taken into account in two situations: that involving a constant value and that involving a random value.

### 5.2. Experimental Analysis

According to the above experimental method, two experiments were conducted.

#### 5.2.1. The First Experiment: *F_N_* is Constant

When *F_N_* is a constant value, considering the effects of the actual noise on the signal, three typical *F_N_* values are used for the experiments, these being 0.1, 0.2, and 0.3, respectively.

The experiments for the three parameters are shown as follows.

- *F_N_* = 0.1

**Figure 6:**
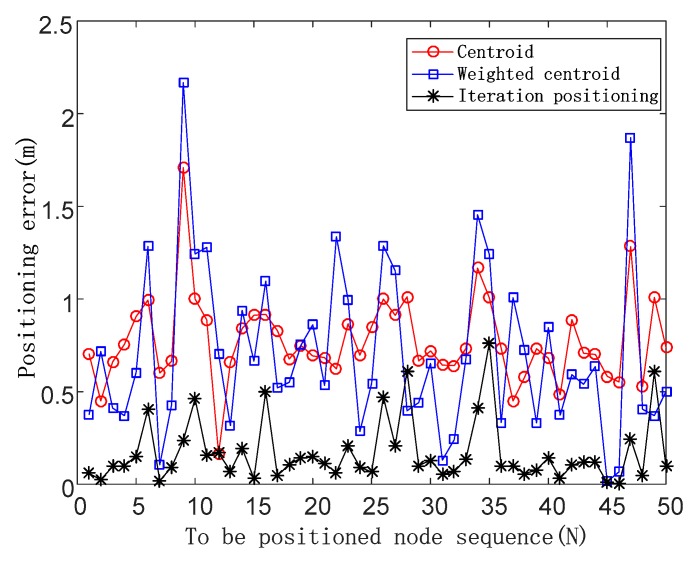
Positioning errors of three positioning algorithms in the case of the noise impact factor of 0.1.

The positioning errors are shown in Figure 6 in the case of the noise impact factor of 0.1.

The *x*-coordinate represents the target node sequence, the unit is the number of nodes, and the *y*-coordinate represents the positioning error, with the unit being m (meters).

When *F_N_* is 0.1, the average positioning errors of the three algorithms are 0.77, 0.70, and 0.17, respectively, and the positioning accuracy of the iterative positioning algorithm is improved by 77.92% and 75.71% compared with the centroid algorithm and the weighted centroid algorithm.

- *F_N_* = 0.2

**Figure 7:**
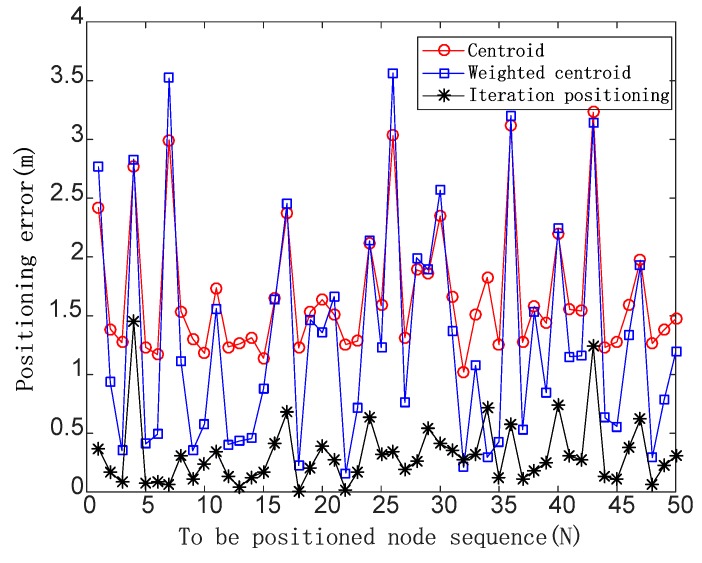
Positioning errors of three positioning algorithms in the case of the noise impact factor of 0.2.

The positioning errors are shown in Figure 7 in the case of the noise impact factor of 0.2.

When *F_N_* is 0.2, the average positioning errors of the three algorithms are 1.68, 1.30, and 0.42, respectively, and the positioning accuracy of the iterative positioning algorithm is improved by 75% and 67.69% compared with the centroid algorithm and the weighted centroid algorithm.

- *F_N_* = 0.3

**Figure 8:**
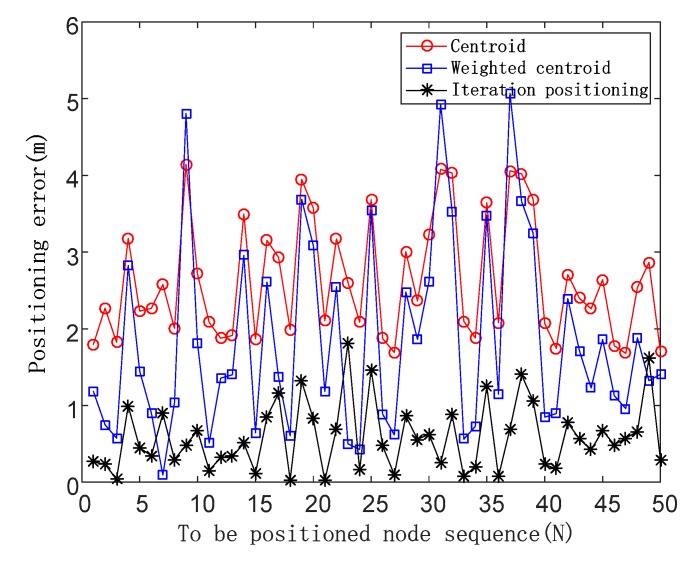
Positioning errors of three positioning algorithms in the case of the noise impact factor of 0.3.

The positioning errors are shown in Figure 8 in the case of a noise impact factor of 0.3.

When *F_N_* is 0.3, the average positioning errors of the three algorithms are 2.63, 1.85, and 0.79, respectively, and the positioning accuracy of the iterative positioning algorithm is improved by 69.96% and 57.29% compared with the centroid algorithm and the weighted centroid algorithm.

#### 5.2.2. The Second Experiment: *F_N_* Is a Random Value

When *F_N_* is an arbitrary value, considering the effect of the actual noise on the signal, the random value is 0.3 times that of the random function.

**Figure 9:**
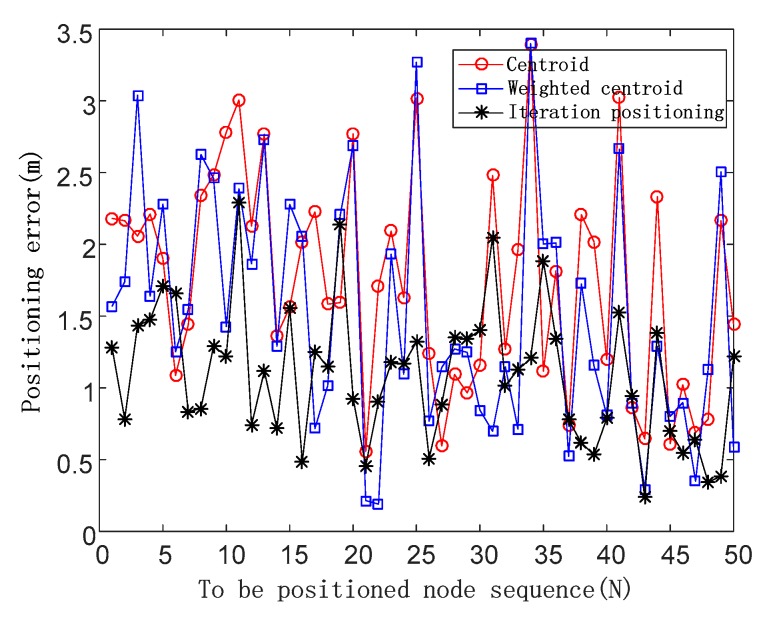
Positioning errors of three positioning algorithms in the case of the noise impact factor of a random value.

The positioning errors are shown in Figure 9 in the case of the noise impact factor of a random value.

In the case of different noise impact factors, the positioning errors of the three positioning algorithms are shown in Table 1.

As can be seen from Table 1, when *F_N_* is constant, the positioning accuracy decreases with the increase of *F_N_*, i.e., the noise has more and more influence on the positioning accuracy; when *F_N_* is a random value, the positioning accuracy of the iterative positioning algorithm is improved by 37.71% compared with the centroid algorithm and the positioning accuracy of the iterative positioning algorithm is improved by 28.76% compared with the weighted centroid algorithm.

**Figure 10:**
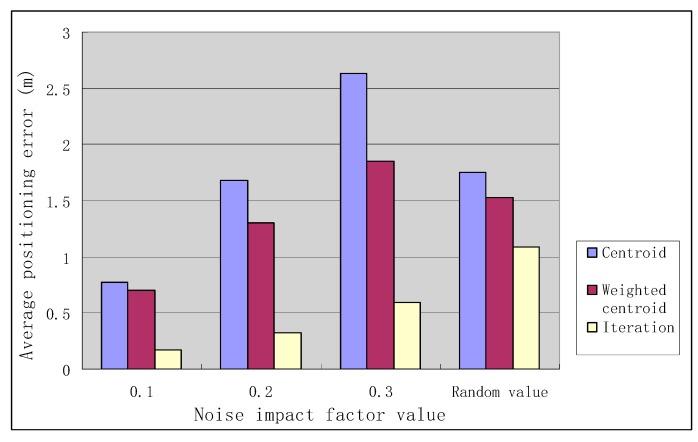
Positioning errors of three positioning algorithms under different noise influence factors.

The positioning errors of the three positioning algorithms for different values of noise impact factors are shown in Figure 10.

As can be seen from Figure 10, the positioning error of the iterative positioning algorithm is smaller than that of the centroid positioning algorithm and the weighted centroid positioning algorithm in the case of different noise impact factors *F_N_*.

## 6. Conclusions

With the development of wireless communication technology, the position information of data is playing an increasingly important role. There are errors in node positioning due to various interferences in the data transmission process. As an alternative to this problem, a node iterative positioning algorithm based on distance correction is proposed in this paper to help users obtain accurate position information. Contributions include the following aspects:

(1) The noise impact factor *F_N_* has been derived based on the original log-distance distribution model, which is used to describe the corresponding relationship between the noise impact factor *F_N_* and the measured distance. Proposing a noise impact factor provides a novel method for analyzing the influence of noise on the distance measurement between nodes in WSN.
(2) The median of the distance deviation coefficient has been constructed to characterize the deviation degree of the whole measured distances and used to correct the range from the last positioning. The triangle centroid localization algorithm has been iteratively conducted based on the adjusted new distance value to improve the node positioning accuracy.

Experimental results show that the node iterative positioning algorithm based on distance correction can reduce the positioning error of unknown target nodes in wireless sensor networks effectively and help users obtain more accurate node coordinates. In the future, based on the node iterative positioning algorithm proposed in this paper, we will move forward with related research including real-time tracking and path planning of moving nodes in WSNs.

## Figures and Tables

**Table 1:** Positioning errors of three positioning algorithms in the case of different noise impact factors.

Noise Impact Factor	Average Positioning Error (m)		
	Centroid	Weighted Centroid	Iterative Positioning
*F_N_* = 0.1	0.77	0.70	0.17
*F_N_* = 0.2	1.68	1.30	0.42
*F_N_* = 0.3	2.63	1.85	0.79
*F_N_* = random	1.75	1.53	1.09
